# Radiation Exposure in Endovascular Infra-Renal Aortic Aneurysm Repair and Factors that Influence It

**DOI:** 10.5935/1678-9741.20160084

**Published:** 2016

**Authors:** Rui Machado, Vitor Miguel Dias Ferreira, Luis Loureiro, João Gonçalves, Pedro Oliveira, Rui Almeida

**Affiliations:** 1 Hospital de Santo António - Centro Hospitalar do Porto, Porto, Portugal.; 2 Instituto de Ciências Biomédicas Abel Salazar (ICBAS), Porto, Portugal.

**Keywords:** Aortic Aneurysm, Abdominal, Endovascular Procedures, Radiation Exposure, Occupational Exposure

## Abstract

**Objective:**

The endovascular repair of aortic abdominal aneurysms exposes the patients and surgical team to ionizing radiation with risk of direct tissue damage and induction of gene mutation. This study aims to describe our standard of radiation exposure in endovascular aortic aneurysm repair and the factors that influence it.

**Methods:**

Retrospective analysis of a prospective database of patients with abdominal infra-renal aortic aneurysms submitted to endovascular repair. This study evaluated the radiation doses (dose area product (DAP)), fluoroscopy durations and their relationships to the patients, aneurysms, and stent-graft characteristics.

**Results:**

This study included 127 patients with a mean age of 73 years. The mean DAP was 4.8 mGy.m^2^, and the fluoroscopy time was 21.8 minutes. Aortic bilateral iliac aneurysms, higher body mass index, aneurysms with diameters larger than 60 mm, necks with diameters larger than 28 mm, common iliac arteries with diameters larger than 20 mm, and neck angulations superior to 50 degrees were associated with an increased radiation dose. The number of anatomic risk factors present was associated with increased radiation exposure and fluoroscopy time, regardless of the anatomical risk factors.

**Conclusion:**

The radiation exposure during endovascular aortic aneurysm repair is significant (mean DAP 4.8 mGy.m^2^) with potential hazards to the surgical team and the patients. The anatomical characteristics of the aneurysm, patient characteristics, and the procedure's technical difficulty were all related to increased radiation exposure during endovascular aortic aneurysm repair procedures. Approximately 40% of radiation exposure can be explained by body mass index, neck angulation, aneurysm diameter, neck diameter, and aneurysm type.

**Table:** 

**Abbreviations, acronyms & symbols**	
ABI	= Aorto-bi-iliac
ALARA	= As Low As Reasonably Achievable
AUI	= Aorto-uni-iliac
BMI	= Body mass index
CT	= Computerized tomography
DAP	= Dose area product
EVAR	= Endovascular aortic aneurysm repair

## INTRODUCTION

The number and complexity of endovascular therapies are increasing in the current treatment of aortic aneurysms. As a result of this conduct change, vascular surgeons are frequently involved in procedures requiring high doses of ionizing radiation, which exposes the surgical team and the patients to potential hazards. This exposure is a necessary and accepted feature of modern vascular surgical practice, yet the training in radiation usage and protection is below ideal, particularly among surgeons^[^^1^^,^^2^^]^.

In addition to the radiation exposure of the patient and the surgical team during the procedure, it is necessary to consider the patient's exposure to radiation during the preoperative study and follow-up through serial computerized tomography (CT) scans. It is estimated that 100 deaths per year occur in the United Kingdom as a direct result of exposure to radiation in diagnosing and treating diseases, and that 700 cancer cases per year result from diagnostic radiography^[^^3^^]^. It is also estimated that cardiology interventional procedures cause 83 cancer cases per 100,000 procedures^[^^3^^]^. The consequences of radiation usage in vascular interventions are less well studied.

The radiation exposure can cause two adverse effects: deterministic effect and stochastic effect. The deterministic effect consists of a direct lesion causing cellular death when a radiation dose exceeds a defined threshold^[^^4^^-^^6^^]^. A transitory skin lesion appears when the 2 Gy dose is exceeded, hair loss occurs above a dose of 3 Gy, skin atrophy and necrosis happen at a dose of 10-12 Gy, desquamation occurs at a dose of 14-18 Gy, and ulceration appears at a dose of 24 Gy or more^[^^4^^,^^7^^]^. There are no such lesions related to the EVAR procedures described, but there is evidence confirming that one third of the patients are exposed to a dose of 2 Gy or more^[^^4^^]^. Additionally, follow-up CT scans or secondary procedures might be required, increasing the risk of the occurrence of direct skin lesions^[^^8^^,^^9^^]^. Often, the signs are subtle and delayed, and the cause-effect relationship may pass unnoticed. The growing utilization of fenestrated or branched endoprosthesis to treat complex aneurysms might increase the radiation usage and the risk of lesion occurrence. A patient's comorbidities, such as diabetes mellitus, conjunctive tissue diseases (systemic erithematosus lupus and sclerodermia), chemotherapy, and previous exposure to radiation also increase the risk of lesion occurrence^[^^4^^]^.

The stochastic effect is related to inducing gene mutation and malignancy. The risk of malignancy increases with cumulative doses of radiation, but it may be induced by any dosage. Therefore, there is not a minimum threshold below which the lesion does not occur, and the severity of the effect is independent of the total radiation dose^[^^5^^,^^6^^]^. It can be evaluated by micronucleus assay of circulating lymphocytes, which is a sensitive marker of biological lesion and of intermediate stages of carcinogenesis^[^^10^^]^. The radiation-induced malignancy is probably of minimal concern; however, younger and fitter patients may have a greater risk and must be informed about the risks and alternatives. Factors associated with an increased cancer risk are young patient age and aneurism neck angulations, which cause difficulties on stent graft placement^[^^11^^]^. A recent debate on whether or not young, good-risk patients should be treated with endovascular abdominal aortic aneurysm repair instead of open surgery shows that it is necessary to understand the late consequences of radiation^[^^12^^]^.

Taking into consideration the deleterious effects of radiation, the EURATOM Directive 97/43 dictates the necessity to protect individuals from radiation, register the quantitative exposure in all procedures, and inform the patients about the risks of exposure^[^^13^^]^. It is essential to know the levels of exposure of each procedure and establish the standard to take actions to diminish the radiation exposure in conformity to the As Low As Reasonably Achievable (ALARA) principle^[^^14^^]^.

This study aims to define the standard of radiation exposure in endovascular aortic aneurysm repair (EVAR) and the factors that influence it.

## METHODS

This study is a retrospective analysis of our prospective database of EVAR that includes treated cases from January 2007 to January 2013, in a university hospital. All EVAR procedures were performed in an operating room using a radiolucent table and Philips C-arm BV Pulsera fluoroscopy. Low-dose fluoroscopy was performed using pulse beam fluoroscopy. All procedures were performed by a team consisting of two senior vascular surgeons. The same surgeon controlled and moved the C-arm, ensuring the homogeneity of the sample. The ALARA principle was applied in all cases. All cases were planned with CT angiogram with reconstruction. Pre-operative C-arm angle was not calculated for all cases.

This study evaluated the indirect measurements of radiation exposure calculated by the C-arm software (Dose area product expressed as mGy.m^2^), fluoroscopy duration, procedure duration, and the patient's age, sex, body mass index (BMI), aneurysm diameter, anatomic type of the endoprosthesis (aorto-biiliac (ABI), aorto-uni-iliac (AUI)), and endoprosthesis fixation (supra-renal or infra-renal). The stent grafts were Gore Excluder (Flagstaff, AZ, USA), Medtronic Talent (Minneapolis, MN, USA), and Medtronic Endurant (Minneapolis, MN, USA). No branched or fenestrated devices were used. Anatomical risk factors that could increase the technical difficulty of the procedure and influence the radiation dose were also registered, including a neck length inferior to 10 mm, a neck angle of more than 50 degrees, a neck diameter of more than 28 mm, a neck calcification of more than 50% of the circumference, a neck thrombus of more than 50% of the circumference, a common iliac diameter more than 20 mm, and great iliac tortuosity. Three groups of anatomical complexity were also defined as: Group 1 having none of the anatomical risk factors listed above; Group 2 having one anatomical risk factor listed above; and Group 3 having two or more anatomical risk factors listed above.

Indirect measurement of the radiation exposure, which was validated in multiple studies, was used as a reliable data source when comparing it with a direct skin dose measurement (peak skin dose) using radiochromic films^[^^4^^,^^5^^]^. The patients' ages were divided into three groups for statistical analysis as follows: less than 70 years old, 70-80 years old, and above 80 years old. The patients' BMIs were subdivided into three categories: less than 25 kg/m^2^, between 25 and 30 kg/m^2^, and above 30 kg/m^2^.

Statistical analysis included a t-test for two independent samples, an analysis of variance in the case of several groups, and a chi-square for comparing proportions concerning categorical variables. Non-parametric tests were also used when normality or homogeneity of variances was not observed. In order to evaluate the relation between exposure to radiation and aneurysm morphological variables together with BMI, several multiple regression models were studied. In order to obtain a normal distribution of the residuals, the dependent variable, exposure to radiation, was log-transformed. The final model included as independent variables, BMI, neck angulation, neck diameter, aneurism diameter and type of aneurysm. BMI was included as continuous variables and the remaining variables were included as binary variables. All of the analyses were performed using IBM SPSS Statistics software, version 22. Statistical significance was set at *P*<0.05.

## RESULTS

The study included 127 patients with an average age of 73 years (minimum 38 years, maximum 92 years). Of these patients, 86.2% were males and 13.8% were females, all with an average BMI of 26.83 (17-43) kg/m^2^. The mean aneurysm diameter was 61 mm (25-106 mm), and the mean neck length was 22.41 mm (5-70 mm). The mean procedure duration was 103 minutes (27-332 minutes), and the mean fluoroscopy time was 20.6 minutes (7.6-64.8 minutes).

The average radiation exposure was 4.8 mGy.m^2^ (standard deviation of 3.2, median 4.0, minimum 0.94, and maximum 15.86). The average fluoroscopy time was 21.8 minutes (standard deviation 11.4, median 19.21, minimum 5.9, and maximum 129.8 minutes). There was a strong correlation between radiation exposure and the fluoroscopy time (Pearson correlation of 0.8). The mean dose area product (DAP) and fluoroscopy time for ABI stent grafts was 4.7 mGy.m^2^ and 22.5 minutes, respectively. For the AUI stent grafts, the mean was 4.4 mGy.m2 and the fluoroscopy time was 18.6 minutes. There was no direct tissue lesions of any level observed that could be associated with deterministic radiation injury.

When the patients were subdivided by age groups, the radiation dose was 4.8 mGy.m^2^, 4.7 mGy.m^2^, and 4.8 mGy.m^2^, and the fluoroscopy time was 20 minutes, 21.9 minutes, and 23.7 minutes for the < 70 years, 70-80 years, and > 80 groups, respectively. Concerning patient sex, the radiation dose was 4.8 mGy.m^2^ for the males and 3.7 mGy.m^2^ for the females; the fluoroscopy time for the males was 22 minutes and was 19.1 minutes for the females. There was no statistical correlation between patient age and sex with the radiation exposure dose and fluoroscopy time (Table 1).

**Table 1:** Radiation exposure and patients' characteristics.

	**DAP (mGym^2^)**		**Fluoroscopy time (minutes)**	
**Age**	**Mean**	**Standard deviation**	**Mean**	**Standard deviation**
< 70 years	4.8	4	20	12.9
70-80 years	4.7	4.2	21.9	9.9
> 80 years	2.8	2.8	23.7	11
**Gender**				
Male	4.8	3.3	22	11.6
Female	3.7	1.7	19.1	8.6
**BMI**				
< 25	4.4	2.7	23.6	11.4
25-30	4.4	3.1	19.1	9.8
> 30	6.7	3.9	13.7	13.7

BMI=body mass index; DAP=dose area product

**Fig. 1:**
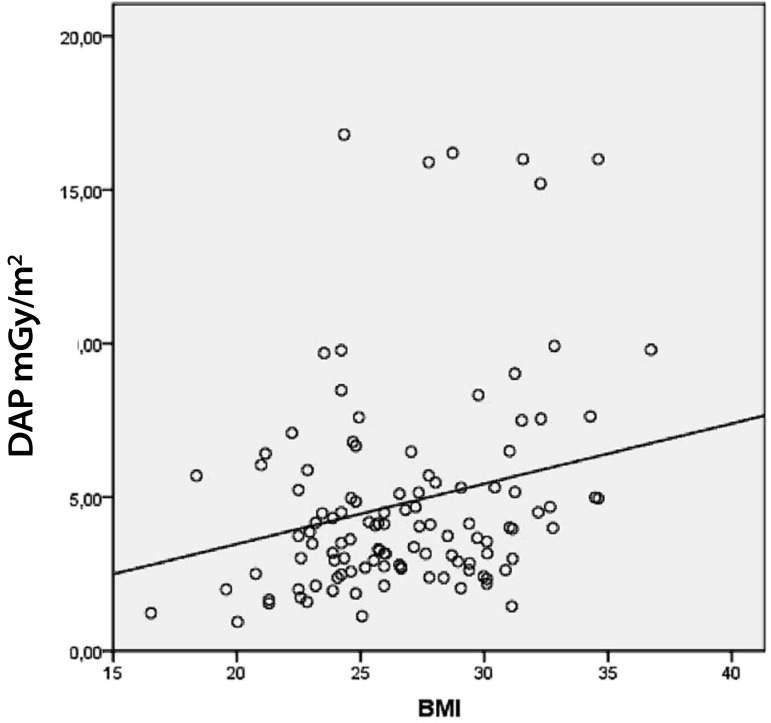
Radiation dose variation and body mass index.

Concerning the radiation exposure and the different BMI groups, there was a statistical correlation between the highest BMI (> 30 kg/m^2^) and increased exposure to radiation, *P*=0.005 (Figure 1). The DAP for the BMI < 25 kg/m^2^ group was 4.4 mGy.m^2^, for the BMI 25-30 kg/m^2^ group was 4.4 mGy.m^2^, and for the BMI > 30 kg/m^2^ group was 6.7 mGy.m^2^ (Table 1). The fluoroscopy time was also influenced by the patient's BMI, with longer exposure times on the patients with a larger BMI (BMI < 25 kg/m^2^, 23.6 minutes; BMI 25-30 kg/m^2^, 19.1 minutes; BMI > 30 kg/m^2^, 25.8 minutes) (Table 1).

Dose radiation and fluoroscopy time were also influenced by the aneurysm diameter. Smaller aneurysms (with a diameter less than 60 mm) were associated with less radiation doses (3.7 *vs.* 5.8 mGy.m^2^, *P*<0.001) and shorter fluoroscopy times (19 *vs.* 24.3 minutes, *P*=0.007) compared with larger aneurysms (with a diameter larger than 60 mm) (Table 2).

**Table 2:** Radiation exposure and aneurysm characteristics.

	**DAP**			**Fluoroscopy time**		
	**Mean**	**Sd**	***P***	**Mean**	**Sd**	***P***
Neck angle <50%	3.7	1.7	<0.001	17.9	17.9	<0.001
Neck angle > 50%	6.6	4.3		28.1	14.3	
Neck calcification < 50%	4.8	3.3	>0.05	21.9	11.7	>0.05
Neck calcification > 50%	5	2.8		19.2	6	
Neck length > 10 mm	4.9	3.3	>0.05	22.2	11.7	>0.05
Neck length < 10 mm	4.5	2.1		19.7	7.7	
Neck diameter < 28 mm	4.6	3.1	=0.003	21.4	11.5	>0.05
Neck diameter > 28 mm	8.5	4.6		27.9	8.6	
Neck thrombus < 50%	4.9	3.3	>0.05	21.9	11.5	>0.05
Neck thrombus > 50%	4.4	2.7		20.6	11.4	
Left common iliac diameter < 20 mm	4.4	2.9	=0.03	20.7	11.1	=0.018
Left common iliac diameter > 20 mm	6.6	3.9		26.6	11.9	
Right common iliac diameter < 20 mm	4.2	2.8	=0.001	20.7	10.9	=0.004
Right common iliac diameter > 20 mm	6.4	3.8		24.8	12.7	
With significant iliac tortuosity	4.8	3.7	>0.05	21.6	13.1	>0.05
Without significant iliac tortuosity	5.6	3.3		24.2	9	
Aneurysm diameter > 60 mm	3.7	1.9	<0.001	19	10.3	=0.007
Aneurysm diameter < 60 mm	5.8	3.8		24.3	11.9	
Aortic aneurysm	4.3	2.8	=0.005	20	10.6	>0.05
Aortic and bilateral iliac aneurysm	7.1	4.9		25.1	14.1	
Aortic and unilateral iliac aneurysm	5.3	2.8		23.5	12.2	
Without anatomic risk factors	3.8	2.7	<0.001	19.1	10.4	=0.009
With 1 anatomic risk factor	4.8	2.7		22.2	10.7	=0.005
With 2 or more anatomic risk factors	6.7	4		26.2	13.2	=0.039

DAP=dose area product; Sd=standard deviation

Regarding the anatomical risk factors previously defined, the radiation dose was increased in patients with a neck angulation superior to 50 degrees (3.7 *vs.* 6.6 mGy.m^2^, *P*<0.001), a neck diameter larger than 28 mm (4.6 *vs.* 8.5 mGy.m^2^, *P*=0.003), and a common iliac diameter larger than 20 mm (4.4 *vs.* 6.6 mGy.m^2^ on the left side, *P*=0.03; 4.2 *vs.* 6.4 mGy.m^2^ on the right side, *P*=0.001). The fluoroscopy time was increased in patients with a neck angulation superior to 50 degrees (17.9 *vs.* 28.1 minutes, *P*<0.001) and in patients with a common iliac diameter bigger than 20 mm (20.7 *vs.* 26.6 minutes on the left side, *P*=0.018; 20.7 *vs.* 24.8 on the right side, *P*=0.004). The neck calcification, neck length, neck thrombus, and increased iliac tortuosity were not associated with statistical significant differences of the radiation dose or fluoroscopy time. When evaluating patients by the number of anatomical complexity risk factors, the radiation exposure was 3.8 mGy.m^2^, 4.8 mGy.m^2^, and 6.7 mGy.m^2^ for no risk factors, one risk factor, and two or more risk factors, respectively. There was a statistical significant difference between the first two groups and the third group (*P*<0.001), demonstrating patients with increased anatomical complexity require more radiation exposure, regardless of which anatomical risk was present. The fluoroscopy time was also associated with the number of anatomical risk factors present with an average of 19.1 minutes, 22.2 minutes, and 26.2 minutes for the zero risk factors, 1 risk factor, and 2 or more risk factors groups, respectively (*P*=0.009) (Table 2).

Concerning the morphology of the aneurysms, the aortic and bilateral iliac aneurysms required higher radiation doses compared to the aortic or aortic and unilateral iliac aneurysms (7.1 *vs.* 4.3 *vs.* 5.3 mGy.m^2^, respectively, *P*=0.005). The different types of stent grafts utilized (e.g., Talent, Excluder, or Endurant) or the presence of supra-renal or infra-renal fixation were not associated with differences on the radiation exposure (Table 2).

The temporal evolution of radiation exposure and fluoroscopy time was erratic during the period of study, with no statistical trend observed.

In order to evaluate the relation between exposure to radiation and aneurysm morphological variables together with BMI, several multiple regression models were studied. The final multiple regression model concerning exposure to radiation is presented in Table 3. Although several different models were studied, the variables included in the final model were: BMI, neck angulation, neck diameter, aneurysm diameter and type of aneurysm. The residual distribution, using as dependent variable the log transformation to radiation exposure, presented an approximated normal distribution. This model exhibited an adjusted R^2^ of approximately 40%. The two most important variables explaining the exposure to radiation were the BMI and neck angulation, as we can observe in Table 3.

**Table 3:** Multiple regression model concerning exposure to radiation.

	**Unstandardized Coefficients**		**Standardized Coefficients**			**95% Confidence Interval for B**	
**Model**	**B**	**Std. Error**	**Beta**	**t**	**Sig.**	**Lower Bound**	**Upper Bond**
(Constant)	-0.197	0.292		-0.673	0.503	-0.776	0.382
BMI	0.049	0.011	0.326	4.440	0.000	0.027	0.071
Neck angulation	0.382	0.096	0.321	3.972	0.000	0.192	0.573
Aneurysm diameter	0.211	0.093	0.186	2.266	0.025	0.026	0.395
Neck diameter	0.365	0.191	0.143	1.916	0.058	-0.012	0.743
Aneurysm type	0.230	0.101	0.164	2.270	0.025	0.029	0.431

## DISCUSSION

Endovascular procedures have become a safe option to treat aortic aneurysms with growing complexity and application. It is of utmost importance to define the standard dose of radiation on endovascular aortic procedures in order to diminish it. There are multiple strategies described in the literature to reduce and limit the use of radiation, such as reducing fluoroscopy use as much as possible, avoiding digital subtraction angiography and magnification, and using proper collimation and radiation control^[^^15^^,^^16^^]^. Image control by the surgeon diminishes the cases of high exposure^[^^17^^]^. All of our cases were performed using a mobile C-arm device controlled by the surgeon in an operating theatre. The exposure of patients and operators to radiation is significantly reduced by routine use of image fusion during standard and complex EVAR and using hybrid fixed-imaging suite^[^^18^^,^^19^^]^. Standardized pre-operative planning of C-arm angle and catheter position with available computer software permits significant decrease of radiation exposure, contrast volume and blood loss^[^^20^^]^.

In this study, and in agreement with the published literature, there was no direct tissue lesions of any level observed. It should be noted that the mean dose exposure (4.8 mGy.m^2^) is above the safe limit and could be associated with transitory skin lesions and hair loss. The reported maximum exposure (15.86 mGy.m^2^) could induce skin atrophy, necrosis, and desquamation. During the procedure, the C-arm is moved for different angulations and incidences, which could diminish the risk of direct tissue lesions.

Concerning the patient characteristics and their effect on the radiation used, the patient age and sex did not influence radiation exposure. A statistically significant correlation was obtained between the radiation exposure dose and the fluoroscopy time with the patient's BMI, in agreement with other case series^[^^4^^,^^9^^,^^21^^-^^23^^]^. In patients with a greater BMI, the X-ray beam must penetrate more tissue to reach the detector, thereby forcing, with automatic exposure control, a greater exposure to obtain an adequate image.

Regarding the characteristics of the aneurysms that were treated, it was found that aortic and bilateral iliac aneurysms, aneurysms larger in diameter, increased neck angles, larger neck diameters, and larger common iliac diameters were associated with more radiation exposure. This was probably related to the increased technical difficulty on these cases. The neck calcification, neck length, neck thrombus, and iliac tortuosity were not associated with statistically significant differences of the radiation dose or fluoroscopy time. Nevertheless, the number of anatomic risk factors present was associated with increased radiation exposure and fluoroscopy time, regardless of which anatomical risk factors were present.

The characteristics of the stent grafts used (*e.g.*, model, anatomic type, supra-renal, or infra-renal fixation) were not related with statistically relevant changes on the radiation dose.

When we compare our mean values of DAP and fluoroscopy time in the ABI group with the results described in the literature (Table 4), the results are comparable with the best published results.

**Table 4:** Literature review.

**First author (year)**	**Procedure (N cases)**	**Fluoroscopy time (minutes)**	**DAP Min (mGy.m^2^)**	**DAP Max (mGy.m^2^)**	**DAP Mean (mGy.m^2^)**	**Mode**
Geijer et al.^21^ (2005)	ABI (24)	28.4	1.66	19.50	7.23	Low dose
Weiss et al.^4^ (2008)	ABI (12)	20.6	5.21	24.54	15.17	NA
Weerakkody et al.^8^ (2008)	ABI (96)	NA	9	65.9	15	NA
Kalef-Ezra et al.^9^ (2009)	ABI (62)	22.6	0.90	28	4.05	Low dose
Maurel et al.^22^ (2012)	ABI (188)	11.2	0.43	28	4.05	Low dose pulsed
Our results	ABI (88)	22.5	1.13	16.2	4.7	Low dose pulsed

ABI=aorto-bi-iliac; AUI=aorto-uni-iliac; DAP=dose area product; NA=not applicable

Concerning the fluoroscopy and procedure times and their comparison with the published literature, the mean values in this study were 21.8 minutes (standard deviation 11.4, median 19.21, minimum 5.9, and maximum 129.8 minutes) and 103 minutes (27-332 minutes), respectively. On the OVER trial^[^^24^^]^ the mean fluoroscopy was 23.0 minutes (17.0-31.0 minutes), and the procedure time was 174 minutes (138-222 minutes). On the DREAM trial^[^^25^^]^ the mean fluoroscopy time was 25 minutes (7-43 minutes), and the procedure time was 135 minutes. On a randomized trial by Becquemin et al.^[^^26^^]^ the mean fluoroscopy time was 16.3 minutes (2.8-29.8 minutes), and the procedure time was 125 minutes (71-179 minutes).

## CONCLUSION

The radiation exposure during EVAR procedures is significant (mean DAP 4.8 mGy.m^2^) with potential hazards to the surgical team and the patients. The anatomical characteristics of the aneurysm (an aneurysm with aortic and bilateral iliac morphology, an aneurysm with a diameter larger than 60 mm, a neck diameter larger than 28 mm, common iliac arteries with a diameter larger than 20 mm, and a neck angulation superior to 50 degrees), the patient characteristics (higher BMI), and the technical difficulty of the procedure (more than 2 anatomic risk factors) were all related to increased radiation exposure during the EVAR procedure. A multiple regression model was developed to predict the relation of radiation exposure with the aneurysm morphological variables and BMI and the two most significant variables related to radiation exposure were BMI and neck angulation. New technology with fusion image and hybrid rooms may decrease the radiation and contrast exposure but are not available in the majority of vascular centres for standard EVAR. The exposure to radiation and the risks it entails should always be part of the proposal of endovascular treatment, particularly in a young patient. The factors listed above, along with the strategies outlined to minimize radiation exposure, must always be present in the planning of using the EVAR procedure to limit the risks for the patient and surgical team.

**Table:** 

**Authors' roles & responsibilities**	
RM	Analysis and/or data interpretation; conception and design study; manuscript redaction or critical review of its content; realization of operations and/or trials; statistical analysis; final manuscript approval
VMDF	Analysis and/or data interpretation; final manuscript approval
LL	Analysis and/or data interpretation; conception and design study; manuscript redaction or critical review of its content; realization of operations and/or trials; statistical analysis; final manuscript approval
JG	Analysis and/or data interpretation; conception and design study; manuscript redaction or critical review of its content; realization of operations and/or trials; final manuscript approval
PO	Analysis and/or data interpretation; conception and design study; manuscript redaction or critical review of its content; statistical analysis; final manuscript approval
RA	Analysis and/or data interpretation; conception and design study; manuscript redaction or critical review of its content; realization of operations and/or trials; statistical analysis; final manuscript approval
